# Determinants of non attendance to mammography program in a region with high voluntary health insurance coverage

**DOI:** 10.1186/1471-2458-8-387

**Published:** 2008-11-13

**Authors:** Magdalena Esteva, Joana Ripoll, Alfonso Leiva, Carmen Sánchez-Contador, Francisca Collado

**Affiliations:** 1 Research Unit, Majorca District Department of Primary Health Care, Balearic Institute of Health, Reina Esclaramunda 9, 07003 Palma de Mallorca, Spain; 2 Breast Cancer Screening Programme, Department of Public Health, Balearic Department of Health, Cecilio Metelo 18, 07003, Palma de Mallorca, Spain

## Abstract

**Background:**

High participation rates are needed to ensure that breast cancer screening programs effectively reduce mortality. We identified the determinants of non-participation in a public breast cancer screening program.

**Methods:**

In this case-control study, 274 women aged 50 to 64 years included in a population-based mammography screening program were personally interviewed. Socio-demographic characteristics, health beliefs, health service utilization, insurance coverage, prior mammography and other preventive activities were examined.

**Results:**

Of the 192 cases and 194 controls contacted, 101 and 173, respectively, were subsequently interviewed. Factors related to non-participation in the breast cancer screening program included higher education (odds ratio [OR] = 5.28; 95% confidence interval [CI95%] = 1.57–17.68), annual dental checks-ups (OR = 1.81; CI95%1.08–3.03), prior mammography at a private health center (OR = 7.27; CI95% 3.97–13.32), gynecologist recommendation of mammography (OR = 2.2; CI95%1.3–3.8), number of visits to a gynecologist (median visits by cases = 1.2, versus controls = 0.92, P = 0.001), and supplemental private insurance (OR = 5.62; CI95% = 3.28–9.6). Among women who had not received a prior mammogram or who had done so at a public center, perceived barriers were the main factors related to non-participation. Among women who had previously received mammograms at a private center, supplemental private health insurance also influenced non-participation. Benign breast symptoms increased the likelihood of participation.

**Conclusion:**

Our data indicate that factors related to the type of insurance coverage (such as prior mammography at a private health center and supplemental private insurance) influenced non-participation in the screening program.

## Background

Breast cancer is the most common cancer among women in developed countries. A woman in the European Union (EU) has an 8% probability of developing breast cancer before the age of 75 and a 2% probability of dying from the disease [1]. Randomized trials have demonstrated that mammography screening reduces breast cancer mortality by 18% to 30% [2]. These findings have led to the implementation of population-based breast cancer prevention programs in many countries [3]. However, high rates of participation among the target population are needed to achieve the reductions in mortality evidenced by clinical trials and organized programs [4,5]. Consequently, the factors that influence women's decisions to participate in organized programs must be identified.

In Spain, population-based programs for breast cancer prevention were gradually introduced during the 1990s. By the end of the decade, organized programs had been established in all regions of the country. Participation in this programs has ranged from 50–80% of the target population [6].

Organized programs were not introduced in the Balearic Islands until 1999. The program target women aged 50 to 64 years and provide mammography screening every two years, with centralized invitations and recruitment of participants. During the first round of the screening program, the rate of attendance was approximately 65%. As Ciatto et al [7] reported, the reasons for not responding to an invitation to mammography screening must be studied in every context. Factors influencing the decision to undergo screening may vary depending on the geographical setting and the organizational aspects of health services in that region. Spain has a national health system with universal access across all regions of the country. However, in the Balearic Islands, 30% of the population has voluntary private health insurance. This proportion increases to 50% among those of the upper social class [8].

Acceptance of mammography may be related to socio-demographic characteristics such as age or social class [9,10] or to health service utilization patterns specifically doctor visits in the previous year, physician recommendation of mammography and access to a regular source of health care [11-14]. Adherence to other preventive practices, benign breast disease or a family history of breast cancer are also factors often associated with the decision to undergo preventive mammography [11-13]. Other reasons for non-participation in screening programs include perceived barriers and a low perception of the breast cancer risk [15,16].

Here, we investigate the factors that influence non-participation in the breast cancer screening program in the Balearic Islands, a region with a high rate of voluntary private health insurance.

## Methods

A case-control study was conducted among women invited to participate in the breast cancer early detection program in Majorca. Cases included women who did not participate in preventive screening and controls included women who chose to participate.

### Study population

The study population were women aged between 50 and 64 years invited to undergo a preventive mammography during the second year of the first round of the breast cancer screening program (1999 to 2000). Women who had undergone a mammogram during the previous year, had been diagnosed with breast cancer, had a mental or physical handicap or had an unknown address were excluded from the study. Women were selected from the program database by simple random sampling (using a table of random numbers). The sample size was calculated based on the assumption that 50% of cases were covered by private and public health insurance. With a power of 0.80 and an alpha error of 0.05, this yielded a sample size of 150 women per group to detect a minimum odds ratio of 2. It was difficult to contact a large number of women; hence, potential subjects were over-recruited by 100%. Replacing subject's criteria was: failure to make contact after five attempts at different times of day, data errors, illness or interview refusal. Interview response rates were higher in the control group than in the case group. Interviews were conducted by trained personnel, using a structured questionnaire and were preceded by a personal letter. None of the subjects refused an interview once the appointment had been scheduled. Interviews were performed at the subjects' homes in 2001 and each lasted an average of 30 minutes.

### Variables

The interview assessed: 1)socio-demographic characteristics -age, education, birthplace and area of residence-, 2)benign breast symptoms that were included in a checklist, 3)family history of breast cancer, 4)preventive activities -mammography during the past two years, Pap smear in the past five years and annual dental check-ups-, 5)attitudes towards mammography among family and friends as suggested by Champion [9], 6)health-service-related variables -mammography recommendation from family doctor or gynecologist, use of health services and type of insurance-, 7)accessibility variables -distance in kilometers to the mammography center and employment status-, 8)reasons for not attending the screening. 9)variables measured by the health belief model scale, which was developed and validated by Champion [17] and subsequently adapted and validated for use in Spain [18].

The health belief model scale has three sub-scales (susceptibility, benefits and barriers) and has been shown to have high reproducibility, internal consistency and construct validity. However, none of the three sub-scales have predictive validity for mammography.

The study protocol was approved by the Research Committee of the Primary Health Care District.

### Statistical analysis

Bivariate analysis was used to assess the relationship between the variables studied and non-participation in the screening program. Crude odds ratio (OR) and confidence intervals (CI) for an alpha error of 5% were calculated by means of logistic regression analysis. Scores on the health belief sub-scales were compared between the two groups using the Mann-Whitney U-test.

Multivariate logistic regression analysis was performed, with the dependent variable defined as non-participation to the mammography appointment. Independent variables showing a statistical significance of <0.25 were selected and backward logistic regression analysis was performed. Possible first-order interactions were assessed in the final model. The statistical significance of each interaction was determined using the likelihood ratio test, with and without the interaction term. Statistical analyses were performed using SPSS software, version 11.5.

## Results

Two random samples of controls and cases were drawn from databases comprising 309 and 326 women, respectively. We were able to contact 194 controls (of the 309, 3 lacked telephones and 112 provided incorrect telephone numbers) and 192 cases (of the 326, 52 lacked telephones and 82 provided incorrect telephone numbers). There were no statistically significant differences in the ages of contacted and non-contacted women. A total of 173 women in the control group were interviewed, with a response rate of 89.2% (173 of 194). Nineteen of the 173 potential controls refused the interview and 2 could not be interviewed for other reasons. A total of 101 cases were interviewed, yielding a response rate of 52.6% (101 of 192). Seven of the 101 potential cases did not meet the inclusion criteria, 56 could not be located, 22 refused the interview and 6 could not be interviewed for other reasons. There were no statistically significant differences in the ages of women who were and were not interviewed.

The characteristics of the cases and controls are summarized in Table 1. Women with higher education levels were five times more likely not to participate in the screening program. No statistically significant differences in program participation were found in relation to age, birthplace or employment status.

**Table 1:** Characteristics of attenders and non-attenders to mammography screening program.

Variables	Cases Non-attenders, n(%) (n = 101)	Controls Attenders, n(%) (n = 173)	Crude OR (95% CI)
Age			
50–54	14 (13.9)	26 (15.1)	1
55–59	36 (35.6)	69 (40.1)	0.96 (0.45–2.08)
60–65**	51 (50.5)	77 (44.8)	1.23 (0.58–2.57)
			
Educational level			
Illiterate-incomplete primary	15 (14.9)	33 (19.2)	1
Primary education	30 (29.7)	72 (41.9)	0.91 (0.43–1.92)
Secondary education	44 (43.6)	61 (35.5)	1.58 (0.77–3.27)
Higher education	12 (11.9)	5 (3.5)	5.28 (1.57–17.68)*
			
Place of birth:			
Balearic islands	60 (59.4)	110 (63.6)	1
Spain	35 (34.7)	58 (33.5)	1.10 (0.65–1.80)
Others	6 (5.9)	5 (2.9)	2.2 (0.64–7.51)
			
Working activity			
Employed	38 (37.6)	58 (33.5)	1
Unemployed-retired-housewife	63 (62.4)	115 (66.5)	0.83 (0.50–1.39)

*p < 0.01 ** some women had 65 at the moment of the interview.

As described in Table 2, family history of breast cancer was not associated with participation in the program, nor was benign breast disease or the degree of mammography approval among family and friends. Furthermore, we did not identify a relationship between program participation and perceived susceptibility to breast cancer, perceived benefits of mammography or perceived barriers to screening.

**Table 2:** Participation according to perceived breast cancer risk, health beliefs and relatives attitudes towards mammography

Variables	Cases Non-attenders n(%) (n = 101)	Controls Attenders n(%) (n = 173)	Crude OR (95% CI)
Family history of cancer			
No	80 (79.2)	142 (82.1)	1
Yes	21 (20.8)	31 (17.9)	1.2(0.64–2.23)
			
Benign breast symptoms			
No	69 (68.3)	103 (59.5)	1
Yes	32 (31.7)	70 (40.5)	0.68(0.41–1.14)
			
Attitudes towards mammography			
			
Spouse			
Neutral/disapproval/disapproval much Approved/	6 (14)	22 (15.9)	1
approved much	37 (86)	116 (84.1)	1.17 (0.44–3.10)
Sons/daughters			
Neutral/disapproval/disapproval much Approved/	3 (7)	8 (6.3)	1
approved much	40 (93)	120 (93.8)	0.88 (0.22–3.51)
Parents			
Neutral/disapproval/disapproval much Approved/	3 (20)	6 (21.4)	1
approved much	12 (80)	22 (78.6)	1.09 (0.23–5.16)
Friend			
Neutral/disapproval/disapproval much Approved/	2 (4.4)	9 (7)	1
approved much	43 (95.6)	119 (93)	1.62 (0.33–7.82)
General practitioner			
Neutral/disapproval/disapproval much Approved/	2 (7.1)	11 (13.6)	1
approved much	26 (92.9)	70 (86.4)	2.04 (0.42–9.84)

	Non-attenders Median (P25–P75)	Attenders Median (P25–P75)	P*

Health Belief Model Scale			
Susceptibility	6.0(3–8)	6.0(3–8)	P = 0.7
Benefits	22.0(20–25)	23.0(21–25)	P = 0.27
Barriers	16.0(12–20)	16.0(14–20)	P = 0.37

* Mann-Whitney U test

Table 3 illustrates the distribution of participants and non-participants, with respect to health behaviors and the use of health services. Non-participants were more likely to have undergone annual dental check-ups and prior mammograms at private facilities, while a history of Pap smears was not associated with participation. Moreover, women with voluntary private health insurance were more likely not to participate in the screening program. Mammography recommendations from gynecologists were associated with a lower probability of participation, whereas receiving a recommendation from a general practitioner (GP) had no influence on attendance. However, the number of visits to a GP within the previous year was significantly higher among participants. In contrast, the number of gynecologist visits was higher among non-participants. There were no differences between the two groups with respect to distance to the mammography center.

**Table 3:** Distribution of attenders and non-attenders to mammography screening program according to health services utilisation.

Variables	Cases Non-attenders, n(%) (n = 101)	Controls Attenders, n(%) (n = 173)	Crude OR (95% CI)
Preventive activities			
Pap test in last 5 years			
No	33(32,7)	69(40.1)	1
Yes	68(67.3)	103(59.9)	1.38 (0.82–2.31)
Annual dental cheks-ups			
No	32(31.7)	79(45.7)	1
Yes	69(68.3)	94(54.3)	1.81 (1.08–3.03)
			
Prior mammography last 2 years			
No prior mammograms	30 (29.7)	97 (56.1)	1
Prior mammograms in public centre	8 (7.9)	48 (27.7)	0.53 (0.23–1.26)**
Prior mammograms in private centre	63 (62.4)	28 (16.2)	7.27 (3.97–13.32)**
			
Type of health insurance coverage			
Public health insurance	29 (28.7)	120 (69.4)	1
Public and private health insurance	72 (71.3)	53 (30.6)	5.62 (3.28–9.6)**
			
Mammography recommended by GPs			
No	81 (80.2)	132 (76.7)	1
Yes	20 (19.8)	40 (23.3)	0.81 (0.44–1.49)
			
Mammography recommended by gynaecologist			
No	26 (25.7)	75 (43.6)	1
Yes	75 (74.3)	97 (56.4)	2.2 (1.30–3.82)**
			P*
			
Visit to general practice(last year)			
Mean (SD)	6.9 (8.6)	8.5(10.1)	0.03
Median (P25–P75)	3 (1–11)	6 (2–12)	
			
Visit to gynaecologist (last 2 years)			
Mean (SD)	1.2 (1.0)	0.9 (1.1)	0.01
Median (P25–P75)	1 (0–2)	1 (0–2)	
			
Distance to mammography(Km)			
Mean (SD)	12.1 (13.0)	13.05 (15.5)	0.90
Median (P25–P75)	10 (1–20)	5 (1–23)	

* Mann-Whitney U test

**p < 0.001

All women provided a reason for not participating, and three women each provided two answers. Among the reasons for not accepting an invitation to screening (Table 4), receiving periodic mammography referrals from a private gynecologist and undergoing a prior mammography that was covered by private health insurance were the most frequent. Failure to receive the letter of invitation was also often mentioned.

**Table 4:** Principal reasons stated for non-attendance to mammography screening program.

Cause of non-attendance	N = 101
No need for screening	
A private gynaecologist makes me periodic gynaecological check-ups	24
I already have had a mammography in a private clinic	30
I already have had mammogram in a public health centre	3
I do not need a mammography, I feel well	5
	
Physical and psychologist barriers	
I did not receive the invitation letter	27
The mammography did not fit well in my agenda	9
I was on travel	3
I forgot the appointment	3
I was afraid	2
I felt lazy	2
	
Number of responses	104

Our data revealed differences between women with supplemental private health insurance and those with only public health insurance (Table 5). A larger proportion of women with supplemental private health insurance had a higher education level, had received frequent gynecologist recommendations for mammography, visited a gynecologist often and had undergone prior mammograms at a private facility.

**Table 5:** Characteristics of women according to type of health insurance coverage.

Variables	Public and private health insurance N (%)	Public health insurance N (%)	Crude OR (95% CI)
Mammography recommendation by gynaecologist.			
No	22 (17.6)	79 (53.4)	1
Yes	103 (82.4)	69 (46.6)	5.30 (3.05–9.40)**
			
Mammography recommendation by GPs			
No	100 (80)	113 (76.4)	1
Yes	25 (20)	35 (23.6)	0.80 (0.45–1.44)
			
Prior mammography last 2 years			
No prior mammograms	32 (26.0)	95 (63.8)	1
Prior mammograms in public centre	8 (6.5)	48 (32.2)	0.49 (0.21–1.15)**
prior mammograms in a private centre	83 (67.5)	6 (4.0)	41.06 (16.36–103.8)**
			
Age			
50–54	15 (12)	25 (16.9)	1
55–59	55 (44)	50 (33.8)	1.83 (0.86–3.86)
60–65	55 (44)	73 (49.3)	1.25 (0.6–2.6)
			
Educational level			
Illiterate-incomplete primary	10 (8.1)	38 (25.5)	1
Primary education	39 (31.5)	63 (42.3)	2.35 (1.05–5.25)***
Secondary education	59 (47.6)	46 (30.9)	4.87 (2.19–10.80)**
Higher education	16 (12.9)	2 (1.3)	30.4 (5.97–154.60)**
			P*
			
Visit to GPs(last year)			
Mean (SD)	6.9 (8.8)	8.9 (10.2)	0.009
Median (P25–P75)	4 (1–10)	6 (2–12)	
			
Visit to gynaecologist (last 2 years)			
Mean (SD)	1.4 (1.1)	0.7 (1.1)	
Median (P25–P75)	2 (0–2)	0 (0–1)	0.000

* Mann-Whitney U test

**p < 0.001

***p < 0,05

Prior mammograms were identified as an effect modifier in our multivariate analysis (Table 6); therefore, separate analyses were performed to assess women who had undergone prior mammograms at a public center, prior mammograms at a private center and those who had not undergone prior mammograms. Among women who had never undergone a prior mammogram or who had done so at a public center, the perceived barriers were the main factors related to non-participation. Among women who had previously undergone mammograms at a private center, supplemental private health insurance influenced non-participation. In this last group, benign breast symptoms increased the likelihood of participation.

**Table 6:** Multiple regression analysis predicting non-participation.

Characteristic	Multivariate OR (CI 95%)*		
	No prior mammograms n = 125	Prior mammograms in a public centre n = 56	Prior mammograms in a private centre n = 87

Benign breast symptoms	0.6 (0.22–1.94)	0.35 (0.05–2.20)	0.33 (0.11–0.99)
Barriers	1.12 (1.03–1.23)	1.19 (1.01–1.41)	0.90 (0.81–1.02)
Public health insurance	1	1	1
Public and private insurance	2.04 (0.77–5.41)	0.36 (0.02–5.88)	14.1 (1.14–174.90)

Age adjusted

*Goodness of fit: no prior mammogram(Hosmer-lemeshow test = 0.701, ROC curve = 0,766); prior mammogram in a public centre(Hosmer-Lemeshow test = 0.101, ROC curve = 0.708); prior mammogram in private centre (Hosmer-Lemeshow test = 0.511, ROC curve = 0.730).

## Discussion

Our findings reveal that private health insurance coverage is most highly associated with non-participation in a screening program. This factor interact with the patterns of preventive health activities shown by women with voluntary private insurance, compared with women who were only covered by public insurance. Women with private insurance tended to be more highly educated, attend gynecologic consultations more often and were more likely to receive mammography recommendations by a gynecologist in a private setting that is, outside of the screening program.

### Limitations of the study

The proportion of successful contacts and interviews was higher among participants than among non-participants in the screening program. Previous studies have also noted a greater difficulty in contacting non-attenders [7,19,20]. This may have resulted in a selection bias in which the non-participation factors were overestimated, because cases who were not interviewed may have provided different reasons for not participating than cases who were interviewed. Some of the non-contacted women may have been seasonal workers because Majorca is the most popular tourist destination in Spain, and may have a different socio-economic status compared with non-seasonal workers. Another limitation inherent in retrospective studies is that attitudes prior to the invitation to a mammography may have influenced subsequent behavior. In addition, undergoing a prior mammogram may have altered attitudes and knowledge. The smaller sample size in this study may have limited to detect any effect of the independent variables such as age and level of education and offers estimated parameters with low precision as can be observed in the multivariate analysis.

### Socio-demographic factors

In this study, highly educated women were less likely to participate in the screening program than those with a lower education level. However, it should be considered that a greater proportion of highly educated women have supplemental private insurance and may therefore be more prone to seek preventive services in the private health care market. Previous studies have suggested that opportunistic programs or self-referral mammographies attract women with medium to high levels of education [11,13,21], whereas organized programs tend to attract women from lower social classes [10,12,20,22]. However, other studies have not reported education-related differences in participation [7,19,20,23-26].

In general, older women are more reluctant to undergo mammography. These women tend to have a lower perception of their breast cancer risk and display more negative attitudes towards screening [9]. At the same time, older women receive less frequent physician recommendations for mammography [27]. This association has predominantly been reported by studies in areas that lack organized programs [9,28,29]. However, differences in participation among the different age groups are reduced in population-based programs, consistent with the results of our study [9,22,25,26,30,31]. Recruitment methods used by organized programs, as well as efforts to ensure equal access for all eligible women, may foster equal access for all age groups.

Working outside the home has been linked to participation in screening programs [11,20,22,27,30]. Similar to results reported by Ciatto et al., [7] our findings reveal no differences in participation between working women and non-working women. It was not possible to assess the differences in participation between Spanish women and women of other nationalities due to the small number of foreign women included in our study.

### Previous experience with breast disease

As previous studies have reported [11,13,26], family history of breast cancer or benign breast disease are not related to participation in the program. However, among women who had previously undergone a mammogram at a private facility, breast symptoms did prompt a response to the invitation, possibly because these women wanted to prevent disease. Consistent with other studies, the distance from a subject's home to the screening center did not seem to influence participation in the program [7,19,23,32]. Our data did not identify a relationship between participation in the program and positive reinforcement from family and friends, as has been described previously [7,9,30]. The low response rate to that question may have reflected limited knowledge regarding relatives' and friends' opinions about the screening program.

### Beliefs

The construct on which health beliefs model is based, the perceived susceptibility to breast cancer and the perceived benefits of and barriers to mammography have been found as predictive of participation. Women who undergo regular mammograms report fewer barriers and perceive more benefits from the screening process [9,16]. Non-participation is more common among women with greater emotional barriers or those who fear that mammography will be painful [32,33]. Some authors [7,33] have reported higher participation rates among women with higher knowledge of the usefulness of mammography. In our study, none of the dimensions of the scale were predictive of attendance. However, the barriers seem to be obstacles for all women except those who had previously undergone a mammogram at a private facility.

### Preventive activities

We observed no differences in participation among women who reported undergoing regular Pap smear tests and those who did not; however, annual dental check-ups were related to non-participation in the program. The inverse correlation between dental check-ups and participation in the program may reflect the fact that dental check-ups are not an included benefit of the public health care system and are therefore more common among women with supplemental private health insurance. Other studies have reported that women who engage in preventive activities are more likely to accept an invitation to a screening program [10,13,20,26,30]. These habits enable women to monitor their own health and offer the opportunity for periodic contact with health professionals.

### Health care providers and use of services

Our results show that frequent visits to a gynecologist and their recommendations for a mammography were associated with non-participation in the screening program. Women who had undergone a preventive mammogram at a private center and those with supplemental insurance were also less likely to participate. At the same time, women with supplemental private insurance were more likely to have attended more gynecologist visits during the previous 2 years and received mammography recommendations. Hence, these data suggest that supplemental private insurance acts as a barrier to participation in population-based breast cancer prevention programs. Women with voluntary private insurance use more preventive services within the private health sector, either of their own volition or on the recommendation of a gynecologist, but do not often participate in public screening programs. Other studies have confirmed lower participation rates among women who visit private gynecologists more frequently or who have undergone mammograms outside of a public screening program [33,35].

In the years since the screening program was introduced, women who usually seek private preventive services have continued to do so and private doctors continue to recommend mammography out of the program. As the public screening program relies primarily on the centralized recruiting of participants, gynecologists in the public sector may not feel as involved in actively recommending mammographies and GPs may be delegating this role to gynecologists [36]. Given that the recommendations of gynecologists and GPs are key factors that increase participation [14,22], these practitioners should be persuaded to actively recommend participation in the population-based program. Family doctors should have a more active role because they have more opportunities to recommend participation in the screening program during numerous patient visits over the course of the year.

Sutton et al [25], reported that women who undergo mammograms outside of a public screening program are, in fact, following screening recommendations and should not be a concern. However, it should be noted that organized programs follow strict quality-control criteria in order to avoid adverse effects and increase detection rates [37]. These practices could not be completely assured in the private sector.

## Conclusion

In summary, we have examined the reasons for non-participation in a population-based breast cancer prevention program in a geographical area where many patients are covered by voluntary private health insurance. These findings have implications for health policies in the region. Health authorities should encourage health professionals in the public sector to promote the benefits of population-based mammography screening programs with established quality-assurance criteria. Guidelines should be disseminated among professionals of the private sector in order to promote participation of women in the organized screening program to ensure optimal prevention. Women should be provided with accurate, balanced information regarding the advantages and risks of participation in organized programs.

## Abbreviations

(EU): European Union; (GP): General Practitioner

## Competing interests

The authors declare that they have no competing interests.

## Authors' contributions

ME and JR designed the study, prepared data collection, did the statistical analysis and wrote the manuscript. AL did the multivariate analysis and participated in the writing of manuscript. CSC and FC participated in the design and data collection and collaborate in writing the discussion and in the review of the manuscript as a whole.

## Pre-publication history

The pre-publication history for this paper can be accessed here:


